# Fusion-ConvBERT: Parallel Convolution and BERT Fusion for Speech Emotion Recognition

**DOI:** 10.3390/s20226688

**Published:** 2020-11-23

**Authors:** Sanghyun Lee, David K. Han, Hanseok Ko

**Affiliations:** 1 Department of Electronics and Electrical Engineering, Korea University, Seoul 136-713, Korea; shlee@ispl.korea.ac.kr; 2 Department of Electrical and Computer Engineering, Drexel University, Philadelphia, PA 19104, USA; dkh42@drexel.edu

**Keywords:** speech emotion recognition, bidirectional encoder representations from transformers (BERT), convolutional neural networks (CNNs), transformer, representation, spatiotemporal representation, fusion model

## Abstract

Speech emotion recognition predicts the emotional state of a speaker based on the person’s speech. It brings an additional element for creating more natural human–computer interactions. Earlier studies on emotional recognition have been primarily based on handcrafted features and manual labels. With the advent of deep learning, there have been some efforts in applying the deep-network-based approach to the problem of emotion recognition. As deep learning automatically extracts salient features correlated to speaker emotion, it brings certain advantages over the handcrafted-feature-based methods. There are, however, some challenges in applying them to the emotion recognition problem, because data required for properly training deep networks are often lacking. Therefore, there is a need for a new deep-learning-based approach which can exploit available information from given speech signals to the maximum extent possible. Our proposed method, called “Fusion-ConvBERT”, is a parallel fusion model consisting of bidirectional encoder representations from transformers and convolutional neural networks. Extensive experiments were conducted on the proposed model using the EMO-DB and Interactive Emotional Dyadic Motion Capture Database emotion corpus, and it was shown that the proposed method outperformed state-of-the-art techniques in most of the test configurations.

## 1. Introduction

In general, emotions are often tightly coupled with social interaction, cognitive processes, and decision making. Human brain processes the multimodalities to extract the spatial and temporal semantic information, that are contextually meaningful to perceive and understand the emotional state of an individual. In particular, human speech contains a wealth of emotional content; therefore, speech emotion recognition (SER) can play an crucial role in communication between humans and computers, as it would aid machines in more accurately predicting speech itself or the speaker’s intention [1]. As such, there is an anticipation that SER may become an important component in social media, and it attracted much interest and research efforts in the area [2,3,4]. For a speech-only system, automatic emotion recognition should understand the fundamental dynamics of emotional cues and be able to identify emotional states from utterances. As recognizing emotions in speech among humans is difficult when cultural differences exist among speakers, the same difficulty remains for SER [5]. Coupled with speech variations among speakers and dynamical features with low saliency, SER is a challenging problem. Previous speech emotion studies have used handcrafted features consisting of low-level descriptors (LLDs), local features such as Mel-frequency cepstral coefficients (MFCCs), energy, or pitch, and have also considered global features by calculating local feature statistics [6,7]. However, extracting handcrafted features requires expensive manual labor and the data quality depends on expert knowledge of labelers. The recent introduction of deep learning models learns relevant features automatically from data without such expert knowledge [8,9,10]. Restricted-Boltzmann-machine (RBM)-based deep neural networks (DNNs) [11,12] or convolutional neural networks (CNNs) [13,14] have shown significant performance improvements over the handcrafted-feature-based methods in prediction. Trigeorgis et al. [9] proposed a hybrid architecture of CNNs extracting local features from a spectrogram and recurrent neural networks (RNNs) taking the output of the CNNs for considering sequentially relevant features from speech. While these deep-learning-based models are effective in the applied scenarios, there are still some significant challenges that these methodologies have yet to overcome. Those issues are as follows:

1. Most of the existing models assume fixed-size input length; however, speech signals are often of variable time durations.
2. Local features extracted by CNNs may not necessarily contain contextually important information on a global scale, as some emotional contents may reside over a lengthy utterance. Previous models of serial fusion that employed RNN structures following the local feature extraction process by CNNs may not be able to capture global scale contextual features [9,15].
3. A new architecture capable of independently capturing both local and global emotional features in lengthy utterances is needed. To address these challenges, we propose an end-to-end technique called “Fusion-ConvBERT”, a parallel network and fusion architecture that automatically captures rich meaning in spatial and temporal features of two models.

Bidirectional encoder representations from transformers (BERT) [16], a recent transformer-based model [17] for natural language processing (NLP), is a pretrained model by unsupervised language models for various NLP tasks. Pretrained models using BERT dominate the NLP world as they have proved highly effective [18,19]. The pretrained models also learn robust phonetic expressions in speech processing tasks, such as speaker recognition and SER [20,21,22,23]. In Fusion-ConvBERT, log mel-spectrograms are extracted from acoustic signals first to be composed as inputs for BERT and CNNs. We employ Mockingjay [21], which is a speech recognition model by pretraining BERT with a large corpus speech data, for fine tuning it for emotion recognition. In the proposed architecture, BERT captures global features associated with lengthy sequential data, as it can maintain bidirectional dependencies, and CNNs are responsible for extracting salient SER features by perceiving local fields of data. In the proposed transformer fusion architecture, BERT and CNNs learn simultaneously. Our major contributions in this paper can be summarized as follows:

- We propose a novel framework to fuse both spatial and temporal representations for SER by leveraging transformer-based Fusion-ConvBERT with pretrained BERT and CNNs, an approach capable of automatically learning feature representations and modeling the temporal dependencies.
- Different from previous serial fusion methods, our method adopts input in multiple features in parallel and simultaneously fuses various emotion details in the transformer layer. The rich interaction that occurs in the fusion allows Fusion-ConvBERT to capture intermediate associations between the local and global patterns and also between different modalities at various representation depths.

We conduct extensive speaker-independent and speaker-dependent experiments, using labeled emotional speech data from the Interactive Emotional Dyadic Motion Capture (IEMOCAP) [24] and the Berlin Database of Emotional Speech (EMO-DB) [25] datasets. To the best of our knowledge, this is the first study in which the BERT model and CNNs are applied to a fusion model, to learn enhanced deep spectrum representations for SER. We demonstrate experimentally that our framework outperforms individual models. For the EMO-DB, our method achieves a weighted accuracy (WA) of 88.43% and an unweighted accuracy (UA) of 86.04% in the speaker-independent experiments. In the speaker-dependent experiments, it achieves a WA of 94.23% and UA of 92.1%. For the IEMOCAP, it achieves a WA of 66.47% and UA of 67.12% in the speaker-independent experiments and a WA of 69.51% and UA of 71.36% in the speaker-dependent ones. The remainder of this paper is organized as follows. In Section 2, existing works related to this research field are presented. In Section 3, we describe the proposed Fusion-ConvBERT framework for speech emotion classification. In Section 4, an experimental analysis comparing the performance of the proposed model on the IEMOCAP and EMO-DB corpora is detailed. Finally, in Section 5, a summary of the study and scope for future work is provided.

## 2. Related Work

SER has attracted considerable attention in digital signal processing research. Recently, researchers have developed various approaches using digital speech signals to identify the emotional conditions of an individual speaker. The focus is on emotion classification by salient acoustic features of speech. Classical machine learning sentiment classifiers include the hidden Markov model (HMM) [26,27], Gaussian mixed model (GMM) [28], and support vector machine (SVM) [29,30]. In general, previous studies have considered both local features (LLDs or MFCCs and energy and pitch) and global features (statistical functionals) [6,31,32]. However, these studies have been based on handcrafted low-level features found to be effective in distinguishing speech emotions. Alternatively, much work has been conducted into applying deep learning to automatically learn useful features relevant to emotion from speech data. Stuhlsatz et al. [12] began with an RBM to preoptimize the network parameters and proceeded with supervised training of a DNN for recognizing speech emotions and improved upon the accuracy of other classifiers (e.g., SVMs). By extracting spectrograms from speech signals and learning hidden time-series information, CNNs can select high-level discriminatory features and recognize the emotional state of the speaker [33,34,35,36]. Traditional SER methods also include bidirectional long-term short-term memory (BLSTM) approaches [37]. Meng et al. [38] proposed a 3-D convolutional network for SER, by combining CNNs with BLSTM. In their model, the 3-D spectral features of segments were used as inputs. Mirsamadi et al. [39] adopted attention mechanisms and feature pooling; thus, the method automatically classified emotions from speech, focusing on the areas of speech signals that were more emotionally prominent. Extracting useful features is essential in good recognition performance, but a fusion method that combines different feature information may further improve classifier results. To this end, Jiang et al. [40] presented an SER fusion method using handcrafted and bottleneck features, whereas Guo et al. [41] and Lim et al. [42] proposed a hybrid CNN-BLSTM model without using any handcrafted features. Although these serial fusion models obtained good results for many speech processing tasks, numerous problems were left unaddressed. For instance, the serial fusion model may not be able to capture global scale emotion contextual features. Furthermore, the deeper the learning model, the easier it falls into overfitting when the database is small. To alleviate these difficulties, we propose a method based on combining a pretrained BERT model and CNN functions to identify salient local spatial features in the SER dataset. The pretrained BERT model prevents overfitting by fine-tuning features of temporal flow over the entire spectrogram frame. Additionally, the model learns by fusing local features extracted from the CNNs and BERT output. The proposed method is shown to be superior to an individual model based on our experiments.

## 3. Proposed Methodology

**Figure 1:**
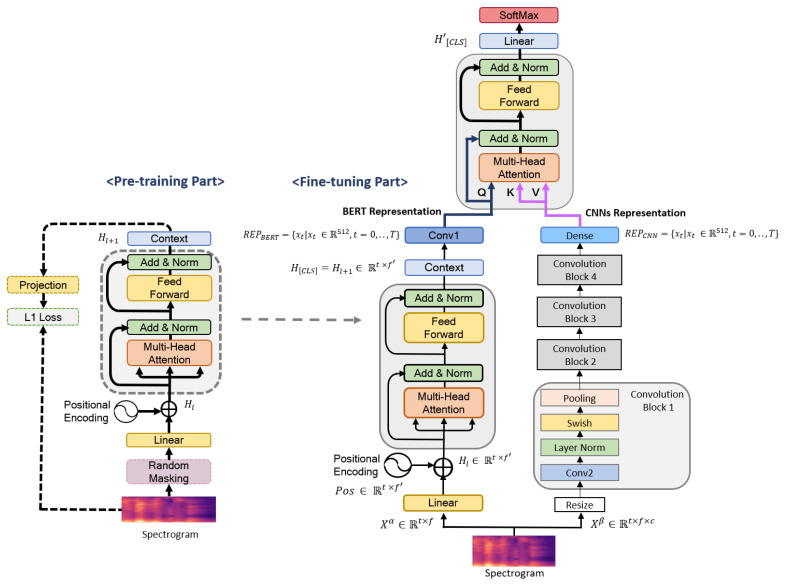
Overview of our proposed Fusion-ConvBERT method for speech emotion recognition. In the pretraining component, a random masking strategy is employed in the spectrogram frame using speech recognition data. Then, unsupervised learning is performed using an L1 loss between the masked and predicted frames. During the fine-tuning process of the BERT model, the spectrogram features are simultaneously input to the pretrained BERT model and CNNs. Meanwhile, Fusion-ConvBERT as a whole is trained by fusion at the transformer; it uses the final hidden-layer features obtained during the fine-tuning of both the CNN- and BERT-based learning components.

In this section, we describe Fusion-ConvBERT, which is formed of two parallel networks (the BERT model and CNNs) and uses log-mel spectrogram as its input (Figure 1). The BERT model extracts speech representations (temporal information) from a spectrogram, whilst the CNNs extract spectrotemporal information. Then, the speech features from these two networks are fused to form a combined spectrotemporal feature vector.

### 3.1. Log-Mel Spectrogram Generation

The first step in the process is to extract log-mel spectrograms as the input to our proposed network. We used the Librosa framework [43] to resample the one-dimensional audio signals received from a microphone to 16 Khz; then, we split them into short frames via a short-time Fourier transform (STFT) [44] using a Hamming window function with a frame length of 25 ms and a rate of 10 ms. Then, the power spectrum for each frame was calculated and transmitted through the mel-filter bank to generate the output $mel_{i,j}\in mel$, where *i* denotes the power spectrum components corresponding to the filter bank and *j* denotes the individual frequency components that span the filter bank region *i*. The relationship between the mel-spectrum (after scaling) and frequency $f^{\prime }$ is expressed via Equation (1):

(1) $$mel=2595\log(1+\frac{f^{\prime }}{700\;\mathrm{Hz}}).$$

The mel-frequency spacing approximates that of the human cochlea, and mel-spectrograms reflect the relative importance of different frequency bands [45]. Then, as shown in Equation (2), the features are normalized using the mean $\mu_{i,j}$ and variance $\sigma_{i,j}$ to obtain $x_{i,j}\in X$ such that −1≤mel≤1.

(2) $$x_{i,j}=\frac{{mel}_{i,j}-\mu_{i,j}}{\sigma_{i,j}}.$$

Equation (3) shows how the log-mel features, calculated from the inputs of the CNNs and BERT model, are reconstructed for each model.

(3) $$X^{\alpha }\in R^{t\times f},\;X^{\beta }\in R^{t\times f\times c}.$$

where *t* denotes the total number of time frames, *f* denotes the number of mel-filter banks (*f* = 160), and *c* denotes the number of feature channels. $X^{\alpha }$ denotes the single channel two-dimensional BERT model input while $X^{\beta }$ represents the CNN input with its channel extended to three dimensions. As the number of frames in the frame-level features varies depending on speech, these features are zero-padded to match the predefined dimension.

### 3.2. CNN Architecture

In this session, we describe the CNN-based architecture for extracting log-mel features for SER. The network is primarily composed of four convolution blocks with indices 1–4.

**Figure 2:**
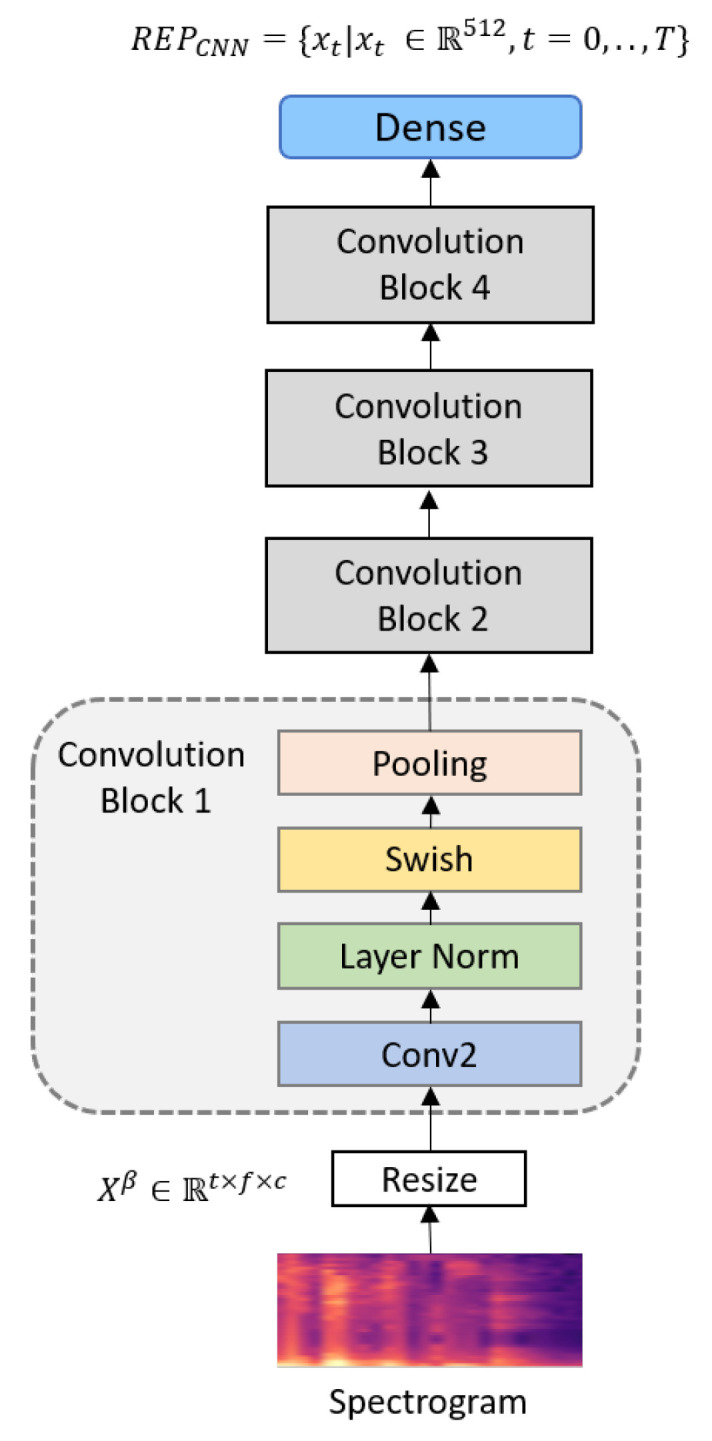
Proposed CNN architecture.

Convolutional blocks are composed of a two-dimensional convolution layer, a layer normalization [46], and a Swish activation [47] in Figure 2. The advantage of the two-dimensional convolution layer is that it can extract local features using the connectivity and shared weights of the spatial information [48,49]. Performing layer normalizations of the convolution layer’s activations at each batch improves the performance and stability of the network. The recently proposed Swish activation is defined as follows:

(4) $$x\varphi \left(\theta x\right)=x\varphi {\left(1+exp\left(-\theta x\right)\right)}^{-1}.$$

where φ(·) denotes the sigmoid function and θ is either a constant or a trainable parameter. The convolution and pooling kernels in each convolution block are two-dimensional. The convolution kernels are of the same size (3×3), stride (1×1), and shape. Convolutional layers can be composed of multiple feature maps. Layers 1–4 contain 64, 128, 256, and 512 convolution kernels, respectively. The parameters of the CNNs are shown in Table 1. Local connections, weight sharing, and downsampling in CNNs can effectively reduce the complexity of the network model and the number of training parameters. The process abstracts input data into high-level feature representations via algorithmic operations between the layers. When only the CNN network is used, the dense output from the fourth layer as shown in Equation (5) is mapped to *K* emotional categories via Softmax.

(5) $$REP_{CNN}=\left\{x_{t}|x_{t}\in R^{512},t=0,\cdots ,T\right\}.$$

### 3.3. Architecture of BERT Model

**Figure 3:**
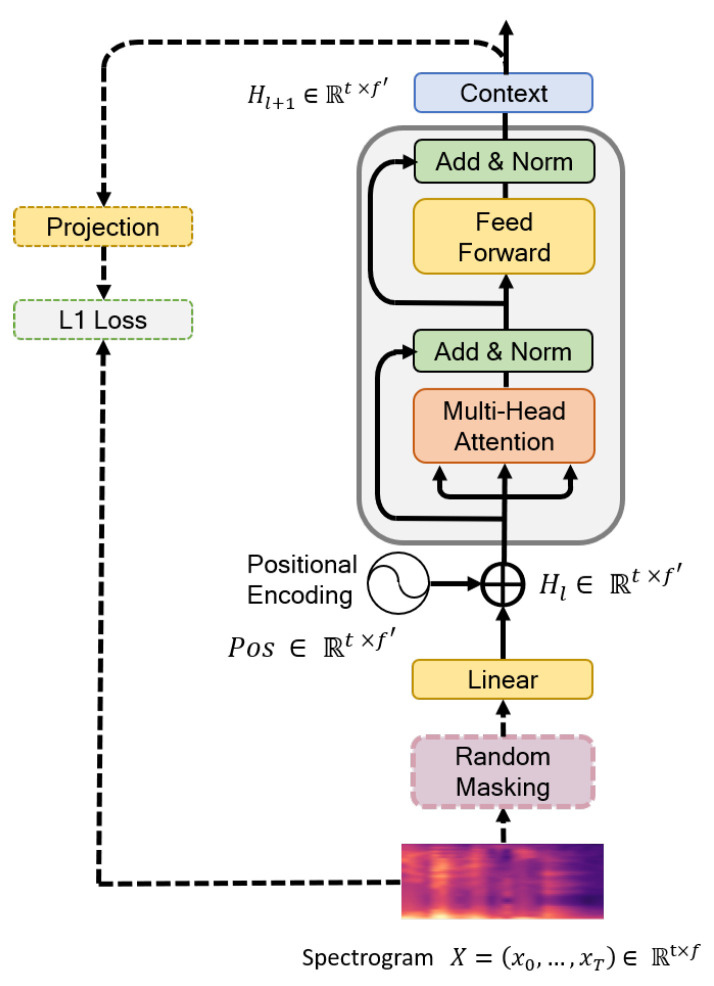
Proposed BERT architecture.

This section introduces the BERT model, which consists of a transformer architecture for extracting temporal features via the self-attention process [16]. Unlike recent language processing models, the BERT model is designed to pretrain deep bidirectional representations from the unlabeled text. Once pretrained on a large language corpus, the BERT model can effectively deliver transfer learning for multiple NLP tasks, such as extracting expressive global features of sentences. The BERT model is commonly trained in a two-step strategy: (1) pretraining and (2) fine-tuning. In our architecture, we begin with the Mockingjay [21] model, which is essentially a BERT pretrained on acoustic data, and proceed to fine-tuning for the emotion classification task. The process starts by taking sequences of mel-feature frames $X=\left(x_{0},\cdots ,x_{T}\right)\in R^{t\times f}$ and passing them through the transformer to produce the learned encoding of BERT representations $H=\left(h_{0},\cdots ,h_{T}\right)\in R^{t\times f}$. In 15% of the frames selected during pretraining, we mask 80% of the time frame, replacing the remainder with 10% of the frames randomly shuffled and 10% unaltered. Given the observed set, the BERT model is trained to minimize reconstruction errors between the prediction and ground-truth frames selected using L1 loss (15%). The BERT structure is presented in Figure 3.

The flow path denoted by dotted lines and the random masking process shown in Figure 3 are used only in the pretraining process while the other elements in the figure were used for both pretraining and fine-tuning. In the BERT architecture, $H_{l}$ denotes an input representation combining a linear layer and position embedding, and it is supplied as the transformer input. Specifically, for the $i_{th}$ head attention in Equation (6), the input layer is based on the dot-product attention mechanism [17] as follows:

(6) $$\begin{array}{cc} att_{i} & =\varepsilon \left(X\right),\\ \; & =softmax\left(\frac{QK^{T}}{\sqrt{m}}\right)V,\\ \; & =softmax\left(\frac{XW_{Q_{i}}W_{K_{i}}^{T}X^{T}}{\sqrt{m}}\right)W_{V_{i}}X. \end{array}$$

We define the query as $Q=XW_{Q}$, the key as $K=XW_{K_{i}}$, and the value as $V=XW_{V_{i}}$, where, $W_{Q_{i}}\in R^{f\times f}$, $W_{K_{i}}\in R^{f\times f}$ and $W_{V_{i}}\in R^{f\times f}$ are weights. Note that $att_{i}$ has the same dimensions as *Q*, *K*, and *V*. The Softmax function in Equation (6) measures the attention given for specific parts of the mel spectrogram, thus $att_{i}$ is features of *V* weighted by attention calculated from *Q* and *K*. The outputs of the *m* attention heads are concatenated together and followed by a linear layer as

(7) $$Linear\left(X\right)=W_{m}{\left[att_{1}\left(X\right),\cdots ,att_{m}\left(X\right)\right]}^{T}+b_{m}.$$

where $W_{m}\in R^{f\times f}$ and $b_{m}$ are learning parameters. The BERT model adds the residual connection from the input to output and then adds the layer normalization [46] as follows:

(8) Z=LayerNorm(X+Linear(X)).

(9) H=LayerNorm(X+Feedforwrad(Z)).

The entire model stacks *L* layers, and the final representation $H_{l+1}$ of the frame is used for the fusion networks.

### 3.4. Fusion-ConvBERT

**Figure 4:**
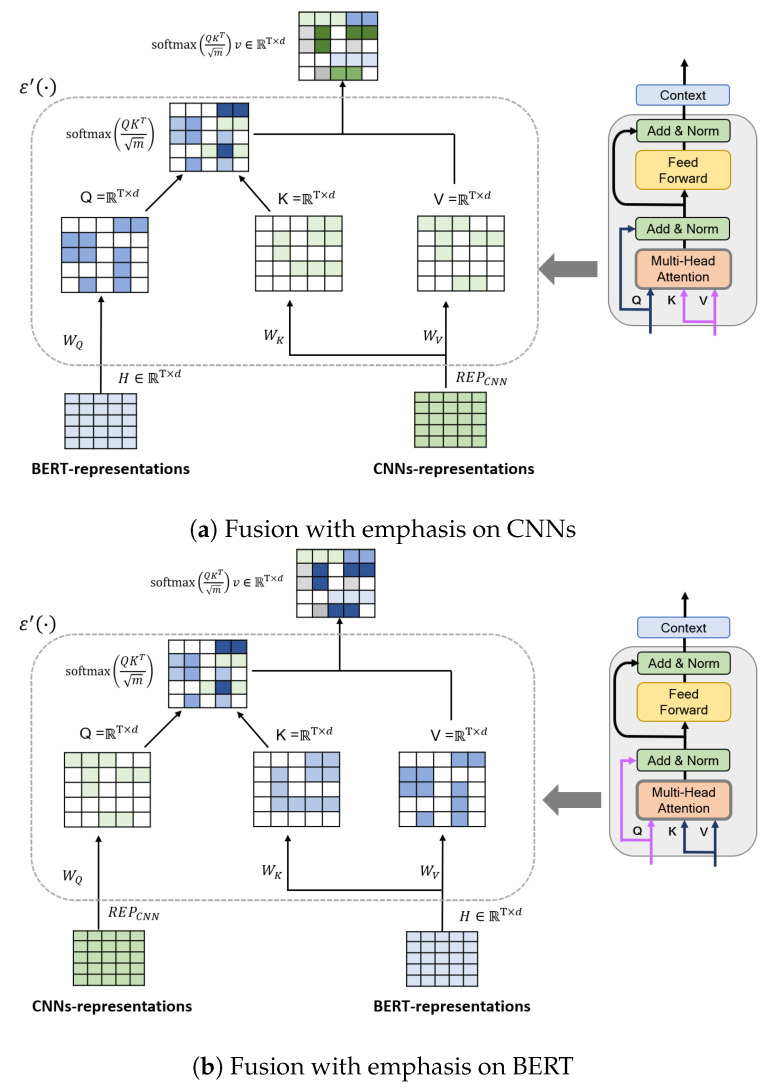
Proposed Fusion-ConvBERT strategy. (**a**), CNN representation is given more emphasis in the attention process; (**b**), BERT representation is given more emphasis in the attention process.

While following the basic layout of NLP encoder transformers, we propose a hybrid transformer model by incorporating a fusion of taking two separate input features in different combinations of *Q*, *K*, and *V*. BERT representations and CNN features are the two input types considered, and one of these was taken as input for *Q* and *K* while *V* was provided from the other representation. As illustrated in Figure 4, an intuitive but general solution that incorporates the two features into the BERT architecture is to directly concatenate the two representations with the final hidden states of the input sequence and then stack additional BERT layers on top, to capture the intermodel interactions between the CNNs and BERT representations. In Equation (5), the output features of the CNNs and $H_{l+1}$ pass through a one-dimensional convolution, to project them into the same feature space. Furthermore, Fusion-ConvBERT applies a transformer to automatically model the rich interactions between the CNNs and BERT model. The $i_{th}$ head attention takes the form of Equation (10) as

(10) $$\begin{array}{cc} att_{i}^{\prime } & =\varepsilon^{\prime }\left(REP_{CNN},H_{l+1}\right)\\ \; & =softmax\left(\frac{H_{l+1}W_{Q_{i}}^{\prime }W_{K_{i}}^{\prime T}REP_{CNN}^{T}}{\sqrt{m}}\right)W_{V_{i}}^{\prime }H_{l+1}. \end{array}$$

where $W_{Q_{i}}^{\prime },W_{K_{i}}^{\prime }$, and $W_{V_{i}}^{\prime }\in R^{f\times f}$ are the parameters. Similarly to the BERT model, the *L* layers are stacked to obtain the final representation by connecting the feedforward layer and residual connections. Finally, we direct the final hidden state of $H_{l+1}^{\prime }$, which captures salient features of different emotions of interest, to the linear function for classification. All modules (i.e., the CNNs and fine-tuned BERT model) are trained simultaneously, to ensure that the model can learn the emotional content of each utterance. As shown in Figure 4, we experimented with two different design configurations of fusion. The first configuration, shown as Figure 4a, applies the CNN-extracted representation for *K* and *V* computations, thus greater attention weight was given to the features obtained by the CNN. Figure 4b depicts the other design option that applies the BERT-extracted representation toward *K* and *V* calculations for giving a larger emphasis to the BERT delivered features. The results of both experiments are discussed in detail in Section 4.2.4.

## 4. Experiments and Results

In this section, we evaluate the proposed system’s effectiveness for SER and compare it against other baseline methods on a publicly available benchmark speech emotion dataset.

### 4.1. Dataset

The Fusion-ConvBERT network was evaluated on two public speech datasets: EMO-DB [25] and IEMOCAP [24]. These two databases contain predetermined sentences spoken by invited actors according to the emotions required. A detailed description of the datasets is given in the following subsections.

#### 4.1.1. EMO-DB Emotion Database

The Berlin EMO-DB was recorded in 2005 and is a German-language SER database. The dataset contains 353 sentences with an average length of approximately 2.7 s recorded at 16 Khz sampling rate. We used seven categories of emotions and they are listed in Table 2.

#### 4.1.2. IEMOCAP Emotion Database

The IEMOCAP database is widely used by researchers in the field of SER. It features two types of dialogue: scripted and improvised. It is an acted English-language SER dataset consisting of five sessions; each session includes two actors (one male and one female), and the database contains 12 h of audio-visual data from all 10 actors recording different emotions, including anger, disgust, fear, sadness, neutral, happiness, and excitement. It is also worth noting that the data distribution of each emotion class is heavily imbalanced. Therefore, following the approach of [50,51], we merged the happiness and excitement utterances into the happiness class. We used four categories of emotions—namely neutral, happiness, sadness, and anger—for training and evaluation. Details are given in Table 3.

### 4.2. Experimental Setup and Evaluation Metrics

We conducted several experiments to evaluate the performance of the proposed framework. These experiments are divided mainly into two categories: speaker-dependent and speaker-independent. Each of these experiments was conducted in two phases: (1) pretraining the BERT setup, and (2) the Fusion-ConvBERT setup. As stated earlier, we applied Mockingjay [21], a speech recognition version of BERT, by pretraining it with the LibriSpeech [52] corpus train-clean-360 containing 1000 h of data. While we followed the main structure of Mockingjay, we found the effect of its downsampling and upsampling parts to be minimum. Thus, for expediency, they were excluded from our architecture. We used the mel-features as inputs to be converted into a high-level representation. In the pretraining phase, we used the same hidden dimension size of H=768, an attention head of A=12, a layer number of L(−layer)=3, and a consecutive masking number of C=7. A total of 500 K epochs were used in the pretraining. In the fusion phase, Fusion-ConvBERT implemented an attention dropout [53] of 0.3 inside the transformer and 20 K training epochs total. The fusion transformer layer was applied with a hidden dimension size of H=120, an attention head of A=6, and a layer number of $L^{\prime }\left(-layer\right)=3$. We used a learning rate of 2e−3 for all models and applied the Adam [54] optimizer and Swish [47] as activation function. We also applied SpecAugment [55] to prevent overfitting. The overall model performance was quantitatively measured using the F1 score, WA, and UA. WA and UA better reflect class-to-class imbalance class averages. We determined WA as the ratio between the correctly classified emotions and the total emotions of the same class. Similarly, UA is the ratio between the correctly predicted emotions and all emotions in the dataset. The metrics are expressed as follows:

(11) $$F1=\frac{2*precision*recall}{precission+recall}.$$

(12) $$WA=\sum_{i}\frac{correct\;utterances}{utterances}.$$

(13) $$UA=\sum_{i}\frac{correct\;utterances\;for\;emotion\;i}{utterances\;of\;emotion\;i}.$$

Furthermore, we performed a detailed ablation study to justify our design choices. First, we built CNN, BERT, and transformer models, to investigate the impacts of spatial and temporal information. The model comparison is summarized as follows: (1) CNNs: we used the proposed CNNs only (see Section 3.2); (2) BERT: we fine-tuned the pretraining BERT model by adding a transformer downstream (see Section 3.3); (3) Transformer: the BERT strategy of masking pretraining was not used and only the transformer encoder architecture was applied; (4) Fusion-ConvBERT: during the fine-tuning process of the BERT model, the pretrained BERT model and the features extracted from CNNs were trained simultaneously in transformer networks. We also compared the fusion performances obtained when using the BERT model for fine-tuning and as a feature extractor in Fusion-ConvBERT. Finally, for Fusion-ConvBERT, we compared the transformer fusion configurations of placing greater emphasis on CNN features versus greater emphasis on BERT features. In other words, the value compares how weights were assigned to spatial features (CNN features) or the temporal features (BERT features). All models were implemented using the Pytorch framework and the suggested models were trained and evaluated on two NVIDIA GeForce GTX 2080TIs.

#### 4.2.1. Speaker-Independent Performance Experiments

We conducted extensive speaker-independent experiments on all the labeled emotional speech data of the EMO-DB and IEMOCAP datasets. The EMO-DB and IEMOCAP corpora contain 10 speakers, and we performed 10-fold cross-validation using a leave-one-out strategy. In each training process, eight speakers from four sessions were used as training data, and the remaining sessions were separated into two parts; thus, eight, one, and one folds were used for the training, development, and testing sets, respectively. Therefore, speaker independence is strictly enforced by testing the model performance by an unseen speaker. As such, the arrangement may also shed some light on how generalizable the models are. We evaluated the proposed system using these datasets and compared several models to verify our design decisions. The comparisons are shown in Table 4 and Table 5.

**Figure 5:**
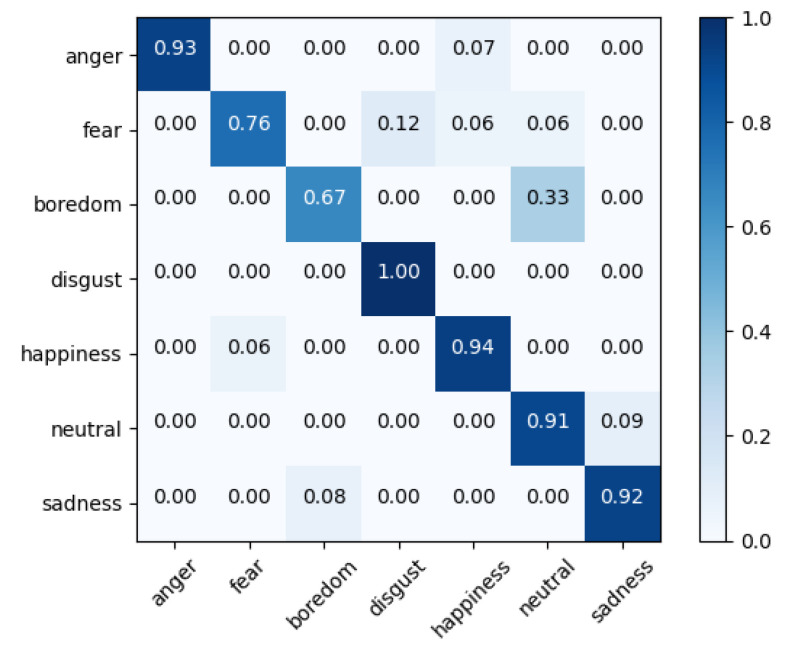
Confusion matrix of speaker-independent emotion prediction on EMO-DB with 86.04% unweighted accuracy overall; the confusion between actual and predicted emotions is shown in the corresponding row.

It is apparent from the tables that Fusion-ConvBERT outperformed all the other models. The highest WA and UA achieved were 88.43% and 86.04%, respectively. For IEMOCAP, a WA and UA of 66.47% and 67.12% were achieved, respectively. Since IEMOCAP data contains more realistic expressions of speaker emotions rather than the EMO-DB data collected from speeches by actors instructed to express content emotions, the model performance is overall lower as can be expected. Models containing the pretrained BERT (BERT or Fusion-BERT) showed higher performances than the other models. From the results, it can be surmised that CNN and the pretrained BERT are extracting information that is mutually exclusive, as the fusion of the two delivers improved performances. It is also observed that the pretraining of BERT delivers better results compared to simply applying the transformer. The proposed architecture of employing a transformer as a fusion structure for combining features taken from CNN and pretrained BERT clearly delivers improved overall performances. The confusion matrix for the proposed Fusion-ConvBERT shows the actually predicted emotions and the model confusion results of each class (Figure 5).

The figure shows improved overall emotion recognition performance for the EMO-DB dataset, however, both “fear” and ”boredom” were identified with accuracies below 80%. While recognition rates for the other five emotions (i.e., “anger”, “disgust”, “happiness”, “neutral”, and “sadness”) exceed 90%; “boredom” was confused with “neutral” due to diversities in how these emotions are expressed among people.

**Figure 6:**
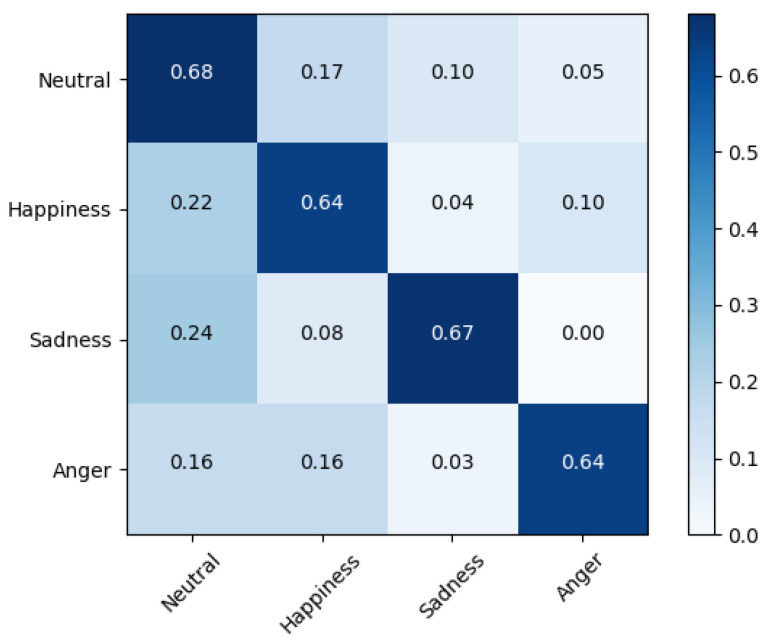
Confusion matrix of speaker-independent emotion prediction on IEMOCAP with 67.12% unweighted accuracy overall; the confusion between actual and predicted emotions is shown in the corresponding row.

Figure 6 shows that on the IEMOCAP dataset, “neutral” was identified with the highest accuracy (68%), “anger” and “happiness” were identified with the lowest accuracy (64%), and both were affected by ”neutral.”

#### 4.2.2. Speaker-Dependent Performance Experiments

In the speaker-dependent experiments, the data were shuffled and the entire set was divided by randomly selecting an 80:20 split ratio for model training and testing, respectively. As before, we investigated the speaker-dependent model using F1-score, WA, and UA. The detailed numerical results for the EMO-DB and IEMOCAP datasets are given in Table 6 and Table 7.

**Figure 7:**
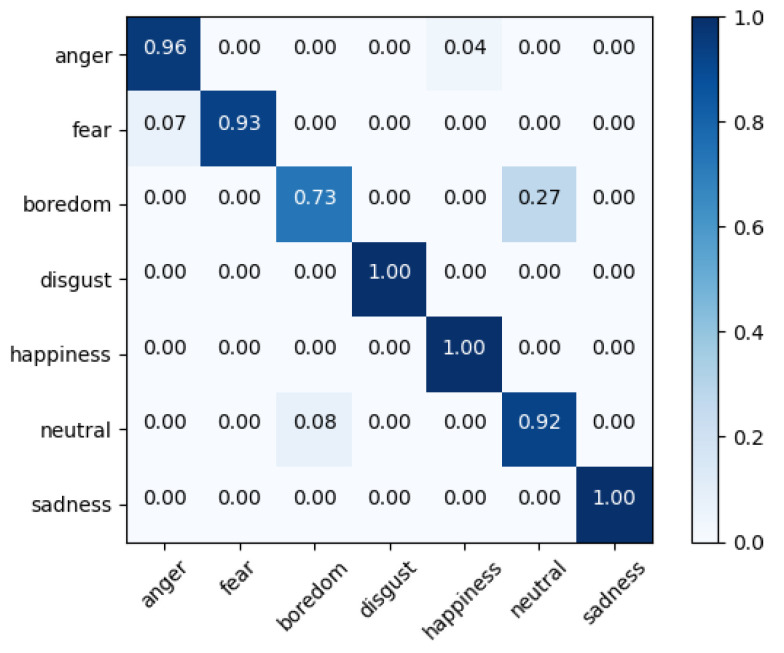
Confusion matrix of speaker-dependent emotion prediction on EMO-DB with 92.1% unweighted accuracy overall; the confusion between actual and predicted emotions in the corresponding row.

**Figure 8:**
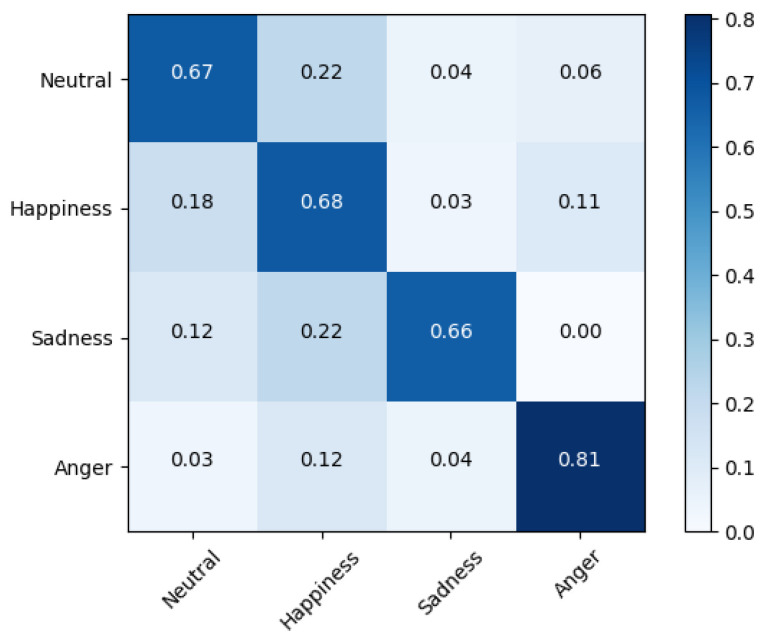
Confusion matrix of speaker-dependent emotion prediction on IEMOCAP with 71.36% unweighted accuracy overall; the confusion between actual and predicted emotions is shown in the corresponding row.

Fusion-ConvBERT again was found superior in this set of experiments. The EMO-DB dataset shows 94.23% WA and 92.1% UA, and the IMEOCPA dataset shows 69.51% WA and 71.36% UA. The confusion matrices for Fusion-ConvBERT in the speaker-dependent experiments are shown in Figure 7 and Figure 8.

In this experiment, the model recognized “disgust”, “happiness, “’ and “sadness” with high accuracy while “boredom” was recognized with a 73% ratio. As in the speaker-independent experiments, ”boredom” was confused with “neutral.”

In the speaker-dependent, IEMOCAP dataset experiments on our network architecture, we found a notable superiority for the “anger” emotion state, with an 81% UA. For “neutral”, “happiness”, and “sadness”, we found almost equal performance results of 67%, 68%, and 66%, respectively. The overall accuracy of the system for speaker-dependent emotion recognition exceeded that of the speaker-independent emotion recognition experiment.

#### 4.2.3. Analysis of Fine-Tuning Effects of Fusion-ConvBERT

As described earlier, Fusion-ConvBERT was first pretrained on the BERT model to compensate for the sparseness of the emotional recognition data. A fusion transformer encoder was added on top of the BERT model, and the overall model is fine-tuned (simultaneously with CNN representation training) as shown in Figure 1. Here, we demonstrate the effectiveness of Fusion-ConvBERT when fused with a pretrained BERT model by comparing performances of two configurations, namely “Fusion-A” and “Fusion-B”. In Fusion-A, learning is applied to the whole network in the fine-tuning process including the pretrained parameters while in Fusion-B the pretrained parameters are frozen in the fine-tuning process.

**Figure 9:**
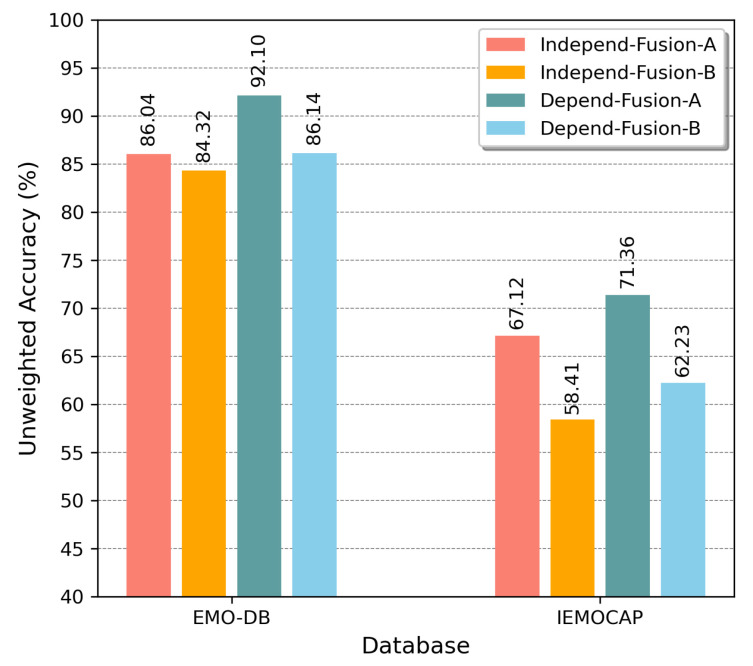
Performance comparison of fine-tuning and feature extractor strategies when training Fusion-ConvBERT with pretrained BERT model, evaluated using the EMO-DB and IEMOCAP datasets with unweighted accuracy overall.

We compared these strategies using both the EMO-DB and IMEOCAP emotion datasets as shown in Figure 9. From the figure, we can see that the Fusion-A outperforms Fusion-B in general. Thus, the value of the fine-tuning method in our architecture is clear.

#### 4.2.4. Analysis of Fusion Strategies

**Figure 10:**
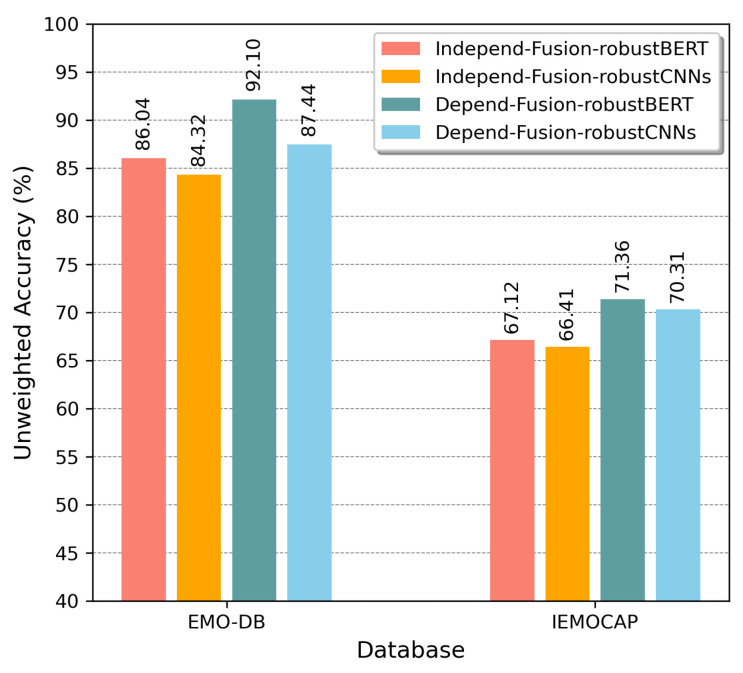
Unweighted overall accuracy comparison of EMO-DB and IEMOCAP according to the two fusion strategies in Fusion-ConvBERT.

We investigated fusion strategies for Fusion-ConvBERT. As shown in Figure 4, in Fusion-ConvBERT, two alternative fusion strategies (BERT-focused and CNN-focused) were applied to the attention mechanism by taking different value combinations of the query, key, and value. Figure 10 shows their experimental performances on the EMO-DB and IEMOCAP datasets.

“Fusion-robustBERT” in Figure 10 indicates the experiments with a query from CNN representation, and the key and value taken from BERT representation, thus greater weight, in essence, is given to the BERT-extracted features. “Fusion-robustCNNs” represents the experiments with query taking the value from BERT representation, and the key and value are from the CNN representation, therefore the CNN features are given greater emphasis. Both the speaker-dependent and speaker-independent tests were conducted using the two fusion configurations and the results are labeled accordingly in the figure. As shown from the figure, the BERT-focused fusion methodology consistently outperforms the CNN-focused fusion. It seems that in the case of emotion recognition, considering the temporal context in large scales, as BERT is capable of, would yield better results compared to placing more emphasis on spectral-temporal correlations and context.

#### 4.2.5. Discussion

SER performance of Fusion-ConvBERT was compared to the existing methods over both the speaker-independent and the speaker-dependent configurations and the results are summarized in Table 8 and Table 9. As most of the existing methods were evaluated in terms of UA scores, only limited performance comparison was possible via WA scores. It can be seen that the approach proposed outperforms existing methods on the EMO-DB and IEMOCAP datasets in all cases except one in the speaker-independent test of EMO-DB dataset.

The reason that the speaker-independent UA score of our model is lower than the one by Guo, L. et al. [41] is that Guo’s model delivered better classification in the Boredom category. It must be noted the dataset on the Boredom class is sparse compared to the other categories, thus the WA score of the same experiment shows our model delivering a better result. It is not clear whether this disparity is due to the sparseness of the data, and warrants future investigation.

## 5. Conclusions

This paper presented Fusion-ConvBERT, a novel fusion network model for SER. Through a series of experiments, the proposed method demonstrated its effectiveness in correctly recognizing the emotional state of a speaker based on the utterance using spectrogram-based features. Two types of features employed here were CNN-based and the BERT-extracted features to consider both spectral-temporal correlations as well as extended temporal contextual information. In the proposed model, these two features were processed simultaneously using a parallel internal structure within the transformer architecture to extract high-level features containing different emotional details. From the ablation study, it was clearly shown that the proposed method delivers improved performance over the individual CNNs, BERT, and transformer models. After verifying the effectiveness of the fusion structure, the model was finalized for performance evaluation. The experiment showed that Fusion-ConvBERT can effectively mine emotional information from spectral and temporal features via an end-to-end technique, and its performance on the EMO-DB and IMEOCAP datasets are superior compared to the state-of-the-art techniques. Fusion-ConvBERT has shown high performance, but one of the areas to be improved for future work is the model complexity resulting in a large number of weight that requires additional computational resources.We will explore simpler structures for combining two different features while retaining the effectiveness of the proposed approach. Additionally, we will investigate improving the model performance in the Boredom class. To improve the Boredom class, we will introduce binary classification to further refine the emotion class and experiment. We expect to achieve real-time speech emotion recognition for human-machine interaction.

## Figures and Tables

**Table 1:** Layer parameters of the CNNs. The output shape is given by the time steps (*T*), mel bins (*F*), and feature maps (*C*). The output *K* denotes the number of emotion targets.

Layers	Output Shape $\left(\mathrm{Time}\;\mathrm{Steps}:T\times \mathrm{Mel}\;\mathrm{Bins}:F\times \mathrm{Feature}\;\mathrm{Maps}:C\right)$	Kernel Size	Stride
Input	T×F×1	−	−
Convolution $\left(1\right)$	T×F×64	3×3	1×1
Pooling $\left(1\right)$	T/4×F/4×64	4×4	4×4
Convolution $\left(2\right)$	T/4×F/4×128	3×3	1×1
Pooling $\left(2\right)$	T/16×F/16×128	4×4	4×4
Convolution $\left(3\right)$	T/16×F/16×256	3×3	1×1
Pooling $\left(3\right)$	T/64×F/64×256	4×4	4×4
Convolution $\left(4\right)$	T/64×F/64×512	3×3	1×1
Pooling $\left(4\right)$	1×1×512	2×2	2×2
Dense	512	−	−
Output	K	−	−

**Table 2:** Emotion class distribution of EMO-DB dataset.

Emotion	Total Utterances	Proportion in (%)
Anger	127	23.74
Fear	69	12.90
Boredom	81	15.14
Disgust	46	8.60
Happiness	71	13.27
Neutral	79	14.77
Sadness	62	11.59
Total	535	100

**Table 3:** Emotion class distribution of IEMOCAP dataset.

Emotion	Total Utterances	Proportion in (%)
Neutral	1708	30.88
Happiness	1636	29.58
Sadness	1084	19.60
Anger	1103	19.94
Total	5531	100

**Table 4:** Performance of the proposed model for speaker-independent emotion recognition on EMO-DB.

Model	F1-Score	WA (%)	UA (%)
CNNs	0.77	79.42	78.12
BERT	0.82	84.77	83.01
Transformer	0.78	80.25	79.1
Fusion-ConvBERT	**0.84**	**88.43**	**86.04**

**Table 5:** Performance of the proposed model for speaker-independent emotion recognition on IEMOCAP.

Model	F1-Score	WA (%)	UA (%)
CNNs	0.63	62.12	64.75
BERT	0.64	64.3	65.11
Transformer	0.63	61.41	64
Fusion-ConvBERT	**0.66**	**66.47**	**67.12**

**Table 6:** Performance of the proposed model for speaker-dependent emotion recognition on EMO-DB.

Model	F1-Score	WA (%)	UA (%)
CNNs	0.84	87.56	85.44
BERT	0.91	91.42	91.12
Transformer	0.86	88.4	87.34
Fusion-ConvBERT	**0.91**	**94.23**	**92.1**

**Table 7:** Performance of the proposed model for speaker-dependent emotion recognition on IEMOCAP.

Model	F1-Score	WA (%)	UA (%)
CNNs	0.62	64.11	66.41
BERT	0.68	70.45	71.12
Transformer	0.66	63.16	65.75
Fusion-ConvBERT	**0.69**	**69.51**	**71.36**

**Table 8:** Speaker-independent and speaker-dependent comparison of the proposed model against baseline methods for the EMO-DB dataset. The optimal results are highlighted in bold. Our model outperforms the current state-of-the-art methods across most evaluation metrics.

Model	Speaker-Indep (WA%)	Speaker-Indep (UA%)	Speaker-Dep (WA%)	Speaker-Dep (UA%)
Guo, L. et al. [41]	87.85	**87.49**	−	87.85
Meng, H. et al. [38]	−	84.99	−	90.37
Chen, M. et al. [15]	−	82.82	−	−
Badshah, A.M. et al. [56]	−	80.79	−	89.46
Jiang, P. et al. [57]	−	84.53	−	86.44
Our model	**88.43**	86.04	**94.23**	**92.1**

**Table 9:** Speaker-independent and speaker-dependent comparison of the proposed model against baseline methods for the IEMOCAP dataset. Optimal results are highlighted in bold. Our model outperforms the current state-of-the-art methods across most evaluation metrics.

Model	Speaker-Indep (WA%)	Speaker-Indep (UA%)	Speaker-Dep (WA%)	Speaker-Dep (UA%)
Guo, L. et al. [41]	56.55	57.99	−	−
Zheng, W. et al. [58]	−	40.02	−	−
Behnke, S. et al. [49]	−	51.24	−	−
Luo, D. et al. [59]	60.35	63.98	−	−
Chen, M. et al. [15]	−	64.74	−	−
Our model	**66.47**	**67.12**	**69.51**	**71.36**
